# New metric of hypoxic dose predicts altitude acclimatization status following various ascent profiles

**DOI:** 10.14814/phy2.14263

**Published:** 2019-10-29

**Authors:** Beth A. Beidleman, Charles S. Fulco, Allen Cymerman, Janet E. Staab, Mark J. Buller, Stephen R. Muza

**Affiliations:** ^1^ U.S. Army Research Institute of Environmental Medicine Natick Massachusetts

**Keywords:** Acute mountain sickness, altitude acclimatization, gradual ascent, hypobaric hypoxia, staging

## Abstract

Medical personnel need practical guidelines on how to construct high altitude ascents to induce altitude acclimatization and avoid acute mountain sickness (AMS) following the first night of sleep at high altitude. Using multiple logistic regression and a comprehensive database, we developed a quantitative prediction model using ascent profile as the independent variable and altitude acclimatization status as the dependent variable from 188 volunteers (147 men, 41 women) who underwent various ascent profiles to 4 km. The accumulated altitude exposure (AAE), a new metric of hypoxic dose, was defined as the ascent profile and was calculated by multiplying the altitude elevation (km) by the number of days (d) at that altitude prior to ascent to 4 km. Altitude acclimatization status was defined as the likely presence or absence of AMS after ~24 h of exposure at 4 km. AMS was assessed using the Cerebral Factor Score (AMS‐C) from the Environmental Symptoms Questionnaire and deemed present if AMS‐C was ≥0.7. Other predictor variables included in the model were age and body mass index (BMI). Sex, race, and smoking status were considered in model development but eliminated due to inadequate numbers in each of the ascent profiles. The AAE (km·d) significantly (*P* < 0.0001) predicted AMS in the model. For every 1 km·d increase in AAE, the odds of getting sick decreased by 41.3%. Equivalently, for every 1 km·d decrease in AAE, the odds of getting sick increased by 70.4%. Age and BMI were not significant predictors. The model demonstrated excellent discrimination (AUC = 0.83 (95% CI = 0.79–0.91) and calibration (Hosmer‐Lemeshow = 0.11). The model provides a priori estimates of altitude acclimatization status resulting from the use of various rapid, staged, and graded ascent profiles.

## Introduction

Rapid ascent to high altitudes >1200 m can negatively impact the health and performance of individuals due to the lowered partial pressure of ambient O_2_ and subsequent arterial hypoxemia (Fulco et al. 1988; American College of Sports Medicine et al. 2017). Altitude acclimatization occurs while living for days to weeks at altitude and encompasses a wide variety of physiologic adaptations that improve oxygen transport to the cells to mitigate the negative effects of arterial hypoxemia (Young and Reeves 2002; Beidleman et al. 1997; Wolfel et al. 2001; Lundby et al. 2004). The summated effect of these adaptations improves physical and cognitive performance and decreases susceptibility to altitude illness (Roach et al. 2002; Wolfel et al. 2001; Banderet et al. 2002; Fulco et al. 2002). When ascent to high altitude outpaces the rate of acclimatization, acute mountain sickness (AMS), a common medical syndrome characterized by headache, loss of appetite, nausea, weakness and dizziness, is more likely to occur (Roach et al. 2002).

Typical altitude acclimatization ascent guidelines that have been followed for many years include the following: (1) ascend gradually if possible, (2) avoid rapid ascent to sleeping altitudes ≥3000 m, (3) don’t ascend more than 300–500 m per day once above 3000 m, (4) for every 1000 m gain in elevation above 3000 m, add an extra day for acclimatization, and (4) stage at altitudes of 1500–2500 m for 4–6 days prior to ascent to a higher altitude (Luo et al. 2013; Roach et al. 2002; Hackett and Roach 2001; Basnyat and Murdoch 2003; Luks et al. 2010; Muza et al. 2010; Luks 2012). In general, faster ascents are more problematic than slower ascents but the optimal ascent rate has never been defined nor has a given ascent threshold (e.g., 300 m per day) been shown to be more efficacious in reducing AMS than a different threshold (e.g., 500 m per day) in a controlled study (Luks 2012). Many combinations of altitude and days at altitude can hypothetically be utilized to induce the same level of acclimatization but there are trade‐offs with each approach (Hackett and Roach 2001; Basnyat and Murdoch 2003; Luks et al. 2010; Luks 2012).

With increased participation in mountain recreation, deployment of US troops to Afghanistan and work sites located at high altitude (Moore 1987; Rodway and Muza 2011; Weng et al. 2013), the need for a quantifiable decision tool to induce acclimatization and effectively manage the risk of AMS has become increasing important. Our laboratory previously developed quantitative models of the incidence and severity of AMS (Beidleman et al. 2013), physical performance degradation (Beidleman et al. 2016), and hematologic responses (Beidleman et al. 2017) following rapid ascent to high altitude. The purpose of this study was to develop a quantitative model of altitude acclimatization status, defined as the presence or absence of AMS after ~24 h of exposure to 4 km, using accumulated altitude exposure (AAE) as a new metric of hypoxic dose. To do this, we utilized multiple logistic regression to determine the relationship between AAE and the prevalence of AMS using a comprehensive database containing individual lowlander subjects with differing physical characteristics that employed various ascent profiles (rapid, gradual, and staged) to the same target altitude of 4 km.

## Methods

### Volunteers and studies

The studies were approved by the Institutional Review Board at the US Army Research Institute of Environmental Medicine and conform to the Declaration of Helsinki. All volunteers provided written and verbal acknowledgement of their informed consent and were made aware of their right to withdraw without prejudice at any time. Investigators adhered to the policies for protection of human subjects as prescribed in Department of Defense Instruction 3216.02 and the research was conducted in adherence with 32 Code of Federal Regulations Part 219.

All volunteers were healthy, well nourished, and physically active lowlanders. Age range was 18 to 66 years. Physical characteristics of the volunteers differed and are provided in Table 1. The present investigation combined individual data from nine studies previously conducted by our research laboratory that employed similar procedures and methods (Table 2) but employed different ascent profiles and therefore accumulated different altitude exposure hours (AAE) prior to ascent to the same target altitude (4 km). All of the studies measured AMS at 4 km after ~24 h of exposure. Thirteen different ascent profiles were utilized in the model and the AAE ranged from 2.105 km∙d to 13.032 km∙d (Table 2). The prevalence of AMS in the nine studies ranged from 0% to 90% depending on the ascent profile. No volunteer participated in more than one study. Eight studies were conducted during field conditions following various ascent profiles to either the summit of Pikes Peak in Colorado (4 km), to an intermediate stopping/sleeping point at 4 km during ascent to Mt. Kilimanjaro, or to an intermediate stopping/sleeping point at 4 km during ascent to Mt. Everest base camp. One study, conducted as part of Operation Everest II, involved slow decompression in a hypobaric chamber to the equivalent altitude of Mt. Everest with an intermediate stopping/sleeping point at 4 km.

**Table 1:** Characteristics of Men (*n* = 147) and Women (*n* = 41) Lowlanders (*n* = 188) utilized in the data set to develop the altitude acclimatization model using various ascent profiles from nine field studies.

Variable	Mean	SD	Min	Max
Age (year)	28	12	18	66
Weight (kg)	75.1	13.2	45.6	135.7
Height (m)	1.75	0.09	1.48	2.00
Body‐mass index (kg/m^2^)	24.4	2.9	18.3	33.9
Accumulated altitude exposure (AAE; km·d)	7.04	3.48	3.02	13.03
Men (%)	78.2			
Acute mountain sickness prevalence (%)	28.7			
Smokers (%)	7.8			
White Caucasians (%)	88.8%			

**Table 2:** Studies from the Mountain Medicine Data Repository utilized to develop the quantitative model of acclimatization status following various ascent profiles.

Study	Location	Ref #	# Subjects	AAE (km·d)	Prior altitude (km)	AMS prevalence (%)	Ascent type	Sex men (%)
1	Air Force Academy/Pikes Peak Laboratory	Muza et al., 2000)	9	4.265	2.0 (4 days)	33.3	Staged	100%
2	Pikes Peak Laboratory	Fulco et al., 2002)	14	3.276	None	71.4	Rapid	100%
3	Pikes Peak Laboratory	Hagobian et al., 2006)	18	2.105	None	77.8	Rapid	100%
4	Air Force Academy/Pikes Peak Laboratory	Beidleman et al., 2009)	11	7.830	2.0 (6 days)	27.3	Staged	100%
5	Pikes Peak Laboratory	Fulco et al., 2011)	9	3.395	None	66.7	Rapid	89%
6	Base Camp Mt. Everest	Unpublished Observations	12	13.032	2.0‐4.0	0.0	Gradual	75%
7	Base Camp Mt. Kilimijaro	Gonzalez et al., 2013)	23	12.222	2.0‐4.0	0.0	Gradual	70%
8	Mt. Everest/Operation Everest II	Sutton, 1992)	8	12.334	2.0‐4.0	0.0	Gradual	100%
9	Pikes National Forest/Pikes Peak Laboratory	Beidleman et al., 2018)	19	5.620	2.5 (2 days)	21.1	Staged	74%
			19	6.620	3.0 (2 days)	21.1	Staged	58%
			16	7.620	3.5 (2 days)	25.0	Staged	81%
			15	8.720	4.0 (2 days)	0.0	Staged	53%
			15	3.020	None	40.0	Rapid	53%

AAE, accumulated altitude exposure, AMS, acute mountain sickness.

None of the volunteers had a previous history of AMS. The model did not include any subjects that took medications such as acetazolamide, dexamethasone, Tylenol, ibuprofen, or gingko biloba. None of the volunteers suffered from any respiratory infections or diarrheal disease nor were they pre‐exposed in a hypobaric or normobaric chamber prior to participating in any of the studies. Considerable efforts were made to sustain caloric balance and euhydration by providing food (similar meals and macronutrient composition) and water ad libitum during data collection before and throughout their altitude exposures. However, body hydration status was not measured in any of the studies. In addition, body weight loss is a normal altitude acclimatization response due to increased energy expenditure, increased water loss, and decreased energy intake at altitude and could not be prevented (Wing‐Gaia 2014). All volunteers participated in physical activity including exercise tests upon arrival at 4 km or graded hiking bouts while staging or ascending to 4 km. Passive versus active ascent was not included as a variable in the model because the volunteers either underwent one or the other and not both for each of the 13 ascent profiles.

### Dependent variable

Altitude acclimatization status, defined as the likely presence or absence of AMS after ~24 h of exposure to 4 km was utilized as the dependent variable in this prediction model. A lower estimated AMS prevalence represented greater altitude acclimatization status. Acute mountain sickness was assessed using the Environmental Symptoms Questionnaire (Sampson et al. 1994). The shortened version of the ESQ, which is a self‐reported 11‐question inventory, was used to quantify a weighted AMS cerebral factor score (AMS‐C) (Beidleman et al. 2007). AMS was judged to be present if an individual’s AMS‐C score was ≥0.7 (Sampson et al. 1994). AMS measurements were made a minimum of 1 h after any type of exercise and were made after the first ~24 h of exposure to 4 km in each study regardless of ascent profile.

### Independent variables

The ascent profile was defined using AAE, a new metric of hypoxic dose. The AAE was utilized in the model as a continuous independent predictor variable by calculating the total altitude exposure prior to one night of sleeping at 4 km. The AAE takes into consideration the days and nights spent at altitude before reaching the final target destination of 4 km and therefore captures the ascent rate. The AAE was calculated in the following manner: (1) if volunteers were flown from sea level to an airport, the hours of decompression in commercial aircraft at 2.4 km (e.g. 8000 feet) were included in the ascent profile, (2) if volunteers were driven to a given altitude over a period of hours, the final altitude reached was divided in half and multiplied by the driving time, (3) given the negligible effect of altitude acclimatization obtained below 1.2 km (American College of Sports Medicine et al. 2017), the dose of altitude exposure was calculated by subtracting 1.2 km from the given exposure altitude, (4) the number of days or partial days spent at various altitudes while ascending was calculated, (5) the one night of sleeping at 4 km was included in the ascent profile in all studies, and (6) a grand sum total of accumulated altitude acclimatization hours was calculated by adding all applicable stages together.

A typical AAE calculation is as follows: (1) six hours flying in a commercial aircraft (e.g. 2.4 km) from Boston, MA to Colorado Springs, CO, (2) 4 days of staging at the Air Force Academy in Colorado Springs, CO at 2.0 km, (3) rapid ascent (2 h) by car to 4.0 km on Pikes Peak, and (4) 20 h of exposure at 4 km prior to an AMS measurement.

$$\text{AAE}\;\left(\text{km}\cdot d\right)\;=\;\left(2.4\;\text{km}-1.2\;\text{km}*\;0.25\;d\right)\;+\;\left(2.0\;\text{km}-1.2\;\text{km}*4\;d\right)\;+\;\left(2.0\;\text{km}-1.2\;\text{km}*0.08\;d\right)\;+\;\left(4.0\;\text{km}-1.2\;\text{km}*0.83\;d\right)\;=\;6.68\;\text{km}\cdot d$$

Other independent variables considered for inclusion in the model were age, sex (men = 0 and women = 1), race (white Caucasian = 0 all others = 1), BMI (kg/m^2^), and smoking status (non‐smokers = 0 and smokers = 1). Following exploratory analysis, sex, race and smoking status were eliminated for inclusion in the model due to inadequate cell representation for each ascent profile. A total of 3 independent variables were considered in the final quantitative model (Age, BMI and AAE). The sample size was based on achieving good predictive ability with three predictor variables (Knofczynski and Mundfrom 2008) and a 40% or greater pseudo R‐square. Based on these values, a sample size of 150 subjects was needed for excellent predictive ability.

## Statistical analyses

### Model development

Multiple logistic regression was conducted using PROC LOGISTIC (SAS 9.4, Cary, NC) to predict the prevalence of AMS at 4.0 km using all independent variables and possible interactions. Best subset regression as well as stepwise, forward, and backward regression models using entry and exit at the 0.10 level were developed with the data set. Many different statistics were utilized to evaluate competing models including the c‐statistic, sensitivity and specificity values, Nagelkerke *R*‐square and the Hosmer‐Lemeshow goodness‐of‐fit test (Hosmer and Lemeshow 2000; LaValley 2008). The cutoff value for the predicted probability was set at 0.50.

### Model diagnostics

Model diagnostics for multiple logistic regression models were performed to compare the data with the fitted model to highlight any discrepancies. There was no evidence of multicollinearity between the predictor values as all tolerance values and condition indices were within the range of acceptable values. Pearson chi‐square statistics was used to identify outliers and influential observations. A change in the Pearson chi‐square statistic >3 and <−3 was considered an outlier (LaValley 2008). Ten subjects had a positive prediction of AMS despite a favorable covariate pattern. After careful inspection, it was determined the data were not erroneous.

### Internal cross‐validation

Cross‐validation of the model was conducted using bootstrap resampling (Efron and Tibshirani 1986, 1993). The procedure was conducted as follows: (1) the model was fitted and parameter estimates determined with the given set of data size *n*; (2) a second data set of size *n* was sampled with replacement from the original data set and called the bootstrap sample; (3) using the bootstrap sample, we refitted the model and obtained the parameter estimates, c‐statistic, pseudo *R*‐square, and correct classification rate; (4) we repeated step 2 1000 times; (5) we took the mean and standard deviation of the 1000 estimates of the parameters, c‐statistic and pseudo *R*‐square. The standard deviation from this process is an estimate of the standard error of these parameters (Fox 2015).

## Results

Table 3 presents the final parameter estimates, standard errors, odd ratios, and *P*‐values for the final quantitative model of altitude acclimatization status, defined as the absence or presence of AMS after spending ~24 h at 4.0 km. The AAE (km·d) (*P* < 0.0001) was the only significant predictors in the model. The final equation was as follows:

$$\text{logit}\;=\;2.188\;+\;\left[\left(-0.5335\right)\;\times \;\left(\text{AAE}\right)\right],\;\text{Probability}\;\left(\text{AMS}\right)\;=\;1/1+e^{-(\text{logit})}$$

**Table 3:** Logistic regression model using accumulated altitude exposure (AAE; km·d) to predict the prevalence of Acute Mountain Sickness (AMS) after ~24 h of exposure to 4300 m and the bootstrap logistic regression model calculated using 1000 samples with replacement.

	*β*	SE (*β*)	OR	95% CI	*P*‐value
AAE (km∙d)	−0.5335	0.0917	0.587	0.486–0.699	0.001
Constant	2.1882	0.4899			
AUC	0.8307	0.0314			
R‐squared	40.0				
% Correct	80.0				
Bootstrap results					
AAE (km∙d)	−0.5444	0.0895			
Constant	2.2226	0.4609			
AUC	0.8151	0.0058			
R‐squared	39.4	0.008			
% Correct	79.1	0.012			

AUC; area under the curve.

**Figure 1:**
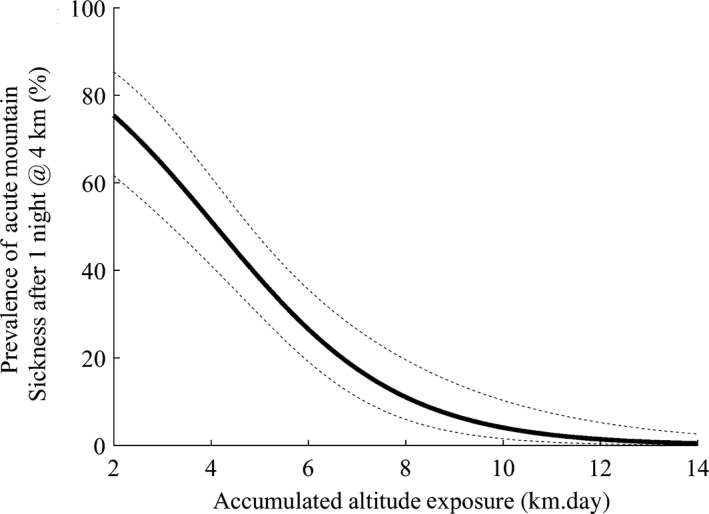
Represents the change in the prevalence of Acute Mountain Sickness (AMS) at 4 km following various ascent profiles inducing various accumulated altitude exposure (AAE; km·d). The equation to calculate the probability of AMS for any given km·d of accumulated altitude exposure (AAE) is as follows: logit = 2.188 + [(−0.5335) x (AAE)], Prob (AMS) = 1/1 + e^−(logit)^. The dotted line represents the 95% confidence interval of the prediction.

Regardless of the starting baseline, for every 1 km·d increase in the AAE, the odds of getting sick decreased by 41.3%. Equivalently, for every 1 km·d decrease in the AAE, the odds of getting sick increased by 72.4%. Age and BMI, were not significant predictors in the model. The percent correct classification was 80% using a 50% cutoff probability to classify an individual with AMS. The Nagelkerke *R*‐square was 40.0%. The area under the curve (AUC) was 0.83 [CI: 0.79–0.91]. Results of the internal cross validation using bootstrap resampling are also presented in Table 3. The difference between the AUC, Nagelkerke *R*‐square, and % correct classification for the original model and 1000 bootstrap samples was <0.2, <0.6 and <0.8% respectively, demonstrating good internal validation (Steyerberg et al. 2010). Figure 1 represents the predicted change in the prevalence of AMS at 4 km following various ascent profiles (and therefore differing AAE) and the 95% confidence interval for the prediction.

## Discussion

This study provides a quantitative model of altitude acclimatization status, defined as the presence or absence of AMS, using a new metric of hypoxic dose. This model provides commanders, search and rescue teams, medical personnel, and coaches with a planning tool to predict the likelihood of developing AMS after ~24 h of exposure to 4 km using different ascent profiles (e.g., rapid, gradual, and staged). Utilizing individual data from nine previous studies following thirteen different ascent profiles to the target altitude of 4 km, we determined that accumulated altitude exposure (AAE; km·d) predicts the likelihood of experiencing AMS after ~24 h of exposure to 4 km. Acclimatization can be obtained using various combinations of altitudes and durations of exposure but is best accomplished at altitudes (e.g., ≤3 km) that do not induce significant AMS and sleep disturbances (Weil 2004; Beidleman et al. 2018). One review also suggested that the overall ascent rate, which AAE captures, may be more important than daily gains in sleeping elevation when acclimatizing to altitude (Luks 2012). In this manner, this quantitative model empowers decision‐makers with the likely AMS risk based on the interaction between the magnitude and duration of altitude exposure prior to ascent. This knowledge can be used to alter the mission or trek plan to reduce the likelihood of experiencing AMS or accept the risk of AMS and plan appropriately either with prophylactic (e.g., acetazolamide) or additional personnel interventions.

By undertaking an adequately slow ascent to high altitude, multiple acclimatization processes such as changes in ventilation, acid‐base balance, sympathetic nervous system activity, and hematologic responses have time to adjust to the hypoxic environment in order to deliver more oxygen to the working cells (Young and Reeves 2002). One study demonstrated that for each additional night spent between Lukla (~2800 m) and Pheriche (~4300 m) on the trek to Everest, which equates to about 1 km∙d of AAE, the risk of AMS was decreased by ~19%. This is lower than the 41% decrease in AMS predicted by this model but the participants in that study were experienced mountaineers that were already partially acclimatized and demonstrated a low incidence of AMS following ascent to 4300 m. Therefore, this model guidance is pertinent to the unexperienced lowlander with little to no mountaineering experience that needs quantitative options for obtaining altitude acclimatization to avoid AMS using gradual or staged ascent to a commonly‐accessed altitude. There are >80 mountain peaks around 4 km in the US alone and another 150 mountain peaks with summits at 4 km and above in other parts of the world (United States Department of the Interior 2001) so this research has wide‐reaching practical applications.

The predictions from this quantitative model agree with some (Beidleman et al. 2009, 2018) but not all (Hansen et al. 1967; Stamper 1980) of the published literature on altitude staging guidelines. Older publications recommend extended staging time (e.g. 4 days to 2 weeks) at lower altitudes (1.6–3.5 km) to reduce symptoms of AMS following ascent to 4 km or above (Luo et al. 2013; Hansen et al. 1967; Stamper 1980). Beidleman et al. (2018), however, recently published a report indicating that staging time can be cut in half from four to two days at 3000–3500 m prior to ascent to 4 km and still achieve a 50% reduction in the prevalence of AMS (80% to 40%). The 95% confidence interval of the model developed in this report indicates that 4–6 km∙d of AAE would be needed to cut the prevalence of AMS to 40% when measured after ~24 h at 4 km. Using the calculation methods described in this paper, both 2 days at 3000 m or 6 days at 2100 m (Beidleman et al. 2009, 2018) would achieve this level of AAE. The model, therefore, confirms recently published guidance on the amount of staging required to reduce the risk of AMS but extends this knowledge to allow varying combinations of altitude exposure and duration to achieve this goal.

Although not specifically modeled in this equation, preacclimatization using intermittent exposures to either hypobaric or normobaric hypoxia has been shown to reduce the incidence of AMS following rapid ascent to 4300 m (Beidleman et al. 2004). An important next step would be to extend this model using altitude preacclimatization hours as a metric to see whether or not the same beneficial reductions in AMS are observed. Age and BMI were not significant predictors in the model. Some have demonstrated a lack of either weight or BMI on the prevalence of AMS (Basnyat et al. 1999; Schneider et al. 2002; Gaillard et al. 2004; Wagner et al. 2008; Beidleman et al. 2013) while others have found that overweight workers were 2–3 times more likely to suffer from AMS than normal weight workers during high altitude exposures between 3500 to 5000 m (Ri‐Li et al. 2003; Wu et al. 2012; Yang et al. 2015). A recent report found that body fatness rather than weight or BMI was associated with the risk of AMS which may explain some of the controversy (Dobrosielski et al. 2017). The association between age and the risk of AMS is also controversial. Some reported that age is not a risk factor for the development of AMS (Schneider et al. 2002; Beidleman et al. 2013; Lawrence and Reid, 2016; Dobrosielski et al. 2017) while others found that older individuals were less likely to suffer from AMS (Maggiorini et al. 1990; Honigman et al. 1993; Gaillard et al. 2004; McDevitt et al. 2014). A recent comprehensive meta‐analysis concluded that there is no association between age and AMS (Wu et al. 2018). Although our model clearly demonstrates that BMI and age are not associated with the development of AMS, more work should be done in this area to clarify the discrepant findings.

In the development of this model, the number of women, non‐whites, and smokers was limited. The cells for the 13 different ascent profiles did not have adequate representation of each subgroup to warrant inclusion of these predictors. While conclusions cannot be made from this research, discrepant findings also exist in the literature for the association between sex and AMS. Some have reported that women are protected against AMS (Hannon 1978; Beidleman et al. 2013) while others have reported either an increased risk of AMS in men or no difference between the sexes (Maggiorini et al. 1990; Basnyat et al. 1999; Schneider et al. 2002; Weng et al. 2013; McDevitt et al. 2014; Lawrence and Reid, 2016). Associations between smoking status and AMS are limited but again the results are controversial with some finding no effect while others report an increased or decreased risk for smokers (Gaillard et al. 2004; Li et al. 2011; Beidleman et al. 2013; McDevitt et al. 2014). Ethnicity has not been studied enough to make any clear conclusions. Two studies have highlighted the importance of controlling for adequate hydration (Luo et al. 2013; Basnyat et al. 1999) when conducting this type of research given that dehydration mimics some of the symptoms of AMS (e.g., headache, fatigue, light‐headedness). Clearly, all of this research points to the need for prospective, randomized, controlled studies with large subject numbers to tease out the impact of demographic characteristics on the risk of AMS. (Luo et al. 2013; Basnyat et al. 1999; Castellani et al. 2010; Beidleman et al. 2013). Examination of genomic and epigenetic factors that may play a role in individual susceptibility to AMS may also prove insightful (MacInnis et al. 2010).

Limitations of this study should be acknowledged. First, acclimatization recommendations are only for ascent to ~4 km and this guidance does not include other altitudes. However, 4 km is an easily accessible altitude within the US and Europe, a convenient stopping point on many high‐altitude treks to Everest and Kilimanjaro, and the location of several high altitude work sites. Second, the number of women, smokers, and races other than white were small in this model. Additional studies should be conducted on these populations to further clarify these demographics and increase the explained variance of the model. Third, AAE was used as a continuous predictor variable in the model based on the fact that 13 different ascent profiles were utilized in model development. Although each ascent profile is actually a discrete time point, the continuous assumption appears reasonable given the range of AAE (e.g., 2–13 km∙d) utilized in model development. Last, although internal validation was conducted using boot‐strap resampling, an external validation study using a completely independent data set should be conducted to verify the generalizability of this model.

## Conclusion

This is the first model that provides a priori estimates of altitude acclimatization status, defined as the presence of absence of AMS at 4 km, following various ascent profiles in unacclimatized lowlanders. The easy‐to‐use model employs a new metric of hypoxic dose, termed accumulated altitude exposure (AAE; km∙d), which predicts altitude acclimatization status following ascent and one night of sleep at 4 km. With increased participation in mountain recreation, deployment of US troops to Afghanistan, and work sites located at high altitude, this model provides a much‐needed quantifiable decision tool that will help prevent or effectively manage AMS prior to ascent to the commonly‐accessed altitude of 4 km.

## Conflict of Interest

No conflict of interest for any author.
